# Bis(azido-κ*N*)bis­[6-(pyridin-2-yl)-1,3,5-triazine-2,4-diamine-κ^2^ *N* ^1^,*N* ^6^]manganese(II)

**DOI:** 10.1107/S160053681201046X

**Published:** 2012-03-17

**Authors:** Kun-Miao Wang, Zhi-Hua Liu, Qi Zheng, Chun-Bo Liu, Ming-Ming Miao

**Affiliations:** a Key Laboratory of Tobacco Chemistry of Yunnan, Yunnan Academy of Tobacco Science, Kunming 650106, People’s Republic of China; b College of Chemical Engineering, Kunming University of Science and Technology, Kunming 650224, People’s Republic of China

## Abstract

In the title complex, [Mn(N_3_)_2_(C_8_H_8_N_6_)_2_], the complete molecule is generated by the application of twofold symmetry, and is in a distorted octa­hedral environment, coordinated by four N atoms of two bidentate 6-(pyridin-2-yl)-1,3,5-triazine-2,4-diamine ligands and two N atoms from two azide anions. The two chelated 6-(pyridin-2-yl)-1,3,5-triazine-2,4-diamine ligands form a dihedral angle 74.75 (5)°. The mononuclear mol­ecules are alternatively linked into layers parallel to the *ac* plane *via* N—H⋯N hydrogen bonds. Adjacent layers are connected into a three-dimensional supra­molecular framework by futher N—H⋯N hydrogen-bonding inter­actions.

## Related literature

For background to pyridyl-substituted diamino­triazine and azide ligands, see: Duong *et al.* (2011 ▶); He *et al.* (2004 ▶); Carranza *et al.* (2008 ▶). For an isotypic Zn^II^ structure, see: Zhao *et al.* (2009 ▶).
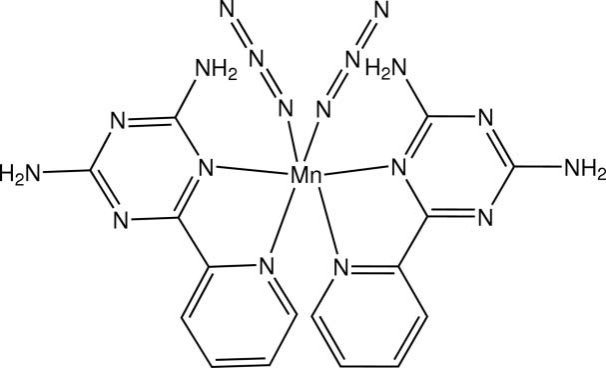


## Experimental

### 

#### Crystal data

[Mn(N_3_)_2_(C_8_H_8_N_6_)_2_]
*M* *_r_* = 515.41
Monoclinic, 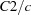

*a* = 18.330 (3) Å
*b* = 14.412 (3) Å
*c* = 9.1915 (17) Å
β = 115.044 (2)°
*V* = 2199.8 (7) Å^3^
*Z* = 4
Mo *K*α radiation
μ = 0.65 mm^−1^
*T* = 293 K
0.17 × 0.11 × 0.10 mm

#### Data collection

Bruker APEXII 1K CCD area-detector diffractometer
Absorption correction: multi-scan (*SADABS*; Sheldrick, 2004 ▶) *T*_min_ = 0.924, *T*_max_ = 0.947
7095 measured reflections
2600 independent reflections
1562 reflections with *I* > 2σ(*I*)
*R*_int_ = 0.056

#### Refinement

*R*[*F* ^2^ > 2σ(*F* ^2^)] = 0.046
*wR*(*F* ^2^) = 0.106
*S* = 1.01
2600 reflections
159 parameters
H-atom parameters constrained
Δρ_max_ = 0.33 e Å^−3^
Δρ_min_ = −0.38 e Å^−3^

### 

Data collection: *APEX2* (Bruker, 2004 ▶); cell refinement: *SAINT* (Bruker, 2004 ▶); data reduction: *SAINT*; program(s) used to solve structure: *SHELXS97* (Sheldrick, 2008 ▶); program(s) used to refine structure: *SHELXL97* (Sheldrick, 2008 ▶); molecular graphics: *SHELXTL* (Sheldrick, 2008 ▶); software used to prepare material for publication: *SHELXTL*.

## Supplementary Material

Crystal structure: contains datablock(s) I, global. DOI: 10.1107/S160053681201046X/pv2519sup1.cif

Structure factors: contains datablock(s) I. DOI: 10.1107/S160053681201046X/pv2519Isup2.hkl

Additional supplementary materials:  crystallographic information; 3D view; checkCIF report

## Figures and Tables

**Table 1:** Hydrogen-bond geometry (Å, °)

*D*—H⋯*A*	*D*—H	H⋯*A*	*D*⋯*A*	*D*—H⋯*A*
N5—H5*B*⋯N7	0.86	2.14	2.986 (3)	166
N5—H5*A*⋯N9^i^	0.86	2.32	2.996 (4)	136
N6—H6*A*⋯N3^ii^	0.86	2.19	3.048 (3)	175
N6—H6*B*⋯N7^iii^	0.86	2.30	3.063 (3)	148

Symmetry codes: (i) 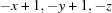; (ii) 
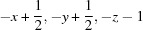
; (iii) 
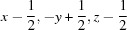
.

**Fig. 1.**
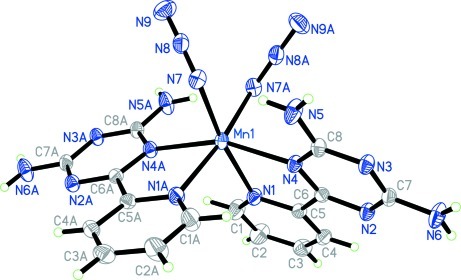
The molecular structure of the title compound with the atom numbering scheme. Displacement ellipsoids are drawn at the 30% probability level. H atoms are presented as small spheres of arbitrary radius. [symmetry code: A -x + 1, y, z - 1/2]

**Fig. 2.**
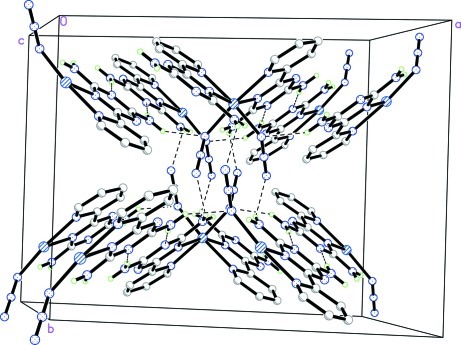
A view of the N—H···N hydrogen bonds (dotted lines) in the crystal structure of the title compound. H atoms non-participating in hydrogen- bonding were omitted for clarity.

## supplementary crystallographic information

### Comment

Pyridyl-substituted diaminotriazine compounds are widely used as ligands in supramolecular chemistry because of their marked ability to associate by forming hydrogen bonds, aromatic interactions, and coordination to metals (Duong *et al.*, 2011). The pseudohalide azides have also been widely used due to their versatile bridging modes. A large number of azide compounds have been synthesized based on two azide coordination modes: 1) the end-on (EO) and 2) the end-to end (EE) (He *et al.*, 2004; Carranza *et al.*, 2008). Herein this article, we report the crystal structure of the title compound, a mononuclear Mn^II^ complex. An isomorphous Zn-complex has been reported (Zhao *et al.*, 2009).

The structure of the title compound (Fig. 1) comprises a mononuclear Mn^II^ complex wherein Mn^II^ lies on an inversion center. The Mn^II^ atom is coordinated by two terminal EO azide anions and two 6-(pyridin-2-yl)-1,3,5-triazine ligands *via* pyridyl and triazine N atoms forming a pseudo-octahedral geometry with Mn—N distances in the range 2.192 (2) to 2.277 (2) Å. The EO azide anions are in a terminal non-linear coordination mode with the Mn^II^ center as is apparent from the Mn1—N7—N8 bond angle of 116.82 (9) °. The 6-(pyridin-2-yl)-1,3,5-triazine ligands are nearly planar, with the largest deviation of any atom from its mean-plane being -0.116 Å for C3. The two chelated diaminotriazine ligands are twisted with respect to each other by a dihedral angle of 74.75 (5) °. The adjacent complex molecules are linked through intermolecular hydrogen bonds N6—H6A···N3 and N6—H6B···N7 between azide groups and diaminotriazine groups (Table 1) forming an infinite hydrogen bonded layer running parallel to the *ac* plane. In addition, hydrogen bonding interactions N5—H5A···N9 connect the neighboring layers to a three-dimensional supramolecular framework (Fig. 2).

### Experimental

A mixture of Mn(CH_3_COO)_2_.2H_2_O (0.1 mmol, 0.025 g), 6-(pyridin-2-yl)-1,3,5-diaminotriazine (0.2 mmol, 0.035 g) in methanol (20.0 ml) was stirred for 40 min and the mixture was filtered, sealed and kept in a dark place at room temperature for several weeks. Block crystals suitable for X-ray diffraction analysis were produced in 10% yield.

### Refinement

The H atoms of pyridine ring and amino groups were generated geometrically and included in the refinement in the riding model approximation with C—H = 0.93 and N—H = 0.86 Å and *U*_iso_(H) = 1.2*U*_eq_(C/N).

### Crystal data

**Table:**

[Mn(N_3_)_2_(C_8_H_8_N_6_)_2_]	*F*(000) = 1052
*M**_r_* = 515.41	*D*_x_ = 1.556 Mg m^−^^3^
Monoclinic, *C*2/*c*	Mo *K*α radiation, λ = 0.71073 Å
Hall symbol: -C 2yc	Cell parameters from 1457 reflections
*a* = 18.330 (3) Å	θ = 2.5–21.6°
*b* = 14.412 (3) Å	µ = 0.65 mm^−^^1^
*c* = 9.1915 (17) Å	*T* = 293 K
β = 115.044 (2)°	Block, pink
*V* = 2199.8 (7) Å^3^	0.17 × 0.11 × 0.10 mm
*Z* = 4	

### Data collection

**Table:**

Bruker APEXII 1K CCD area-detector diffractometer	2600 independent reflections
Radiation source: fine-focus sealed tube	1562 reflections with *I* > 2σ(*I*)
Graphite monochromator	*R*_int_ = 0.056
phi and ω scans	θ_max_ = 28.4°, θ_min_ = 1.9°
Absorption correction: multi-scan (*SADABS*; Sheldrick, 2004)	*h* = −21→24
*T*_min_ = 0.924, *T*_max_ = 0.947	*k* = −19→17
7095 measured reflections	*l* = −11→11

### Refinement

**Table:**

Refinement on *F*^2^	Primary atom site location: structure-invariant direct methods
Least-squares matrix: full	Secondary atom site location: difference Fourier map
*R*[*F*^2^ > 2σ(*F*^2^)] = 0.046	Hydrogen site location: inferred from neighbouring sites
*wR*(*F*^2^) = 0.106	H-atom parameters constrained
*S* = 1.01	*w* = 1/[σ^2^(*F*_o_^2^) + (0.0378*P*)^2^] where *P* = (*F*_o_^2^ + 2*F*_c_^2^)/3
2600 reflections	(Δ/σ)_max_ < 0.001
159 parameters	Δρ_max_ = 0.33 e Å^−^^3^
0 restraints	Δρ_min_ = −0.38 e Å^−^^3^

### Special details

**Table:**

Geometry. All e.s.d.'s (except the e.s.d. in the dihedral angle between two l.s. planes) are estimated using the full covariance matrix. The cell e.s.d.'s are taken into account individually in the estimation of e.s.d.'s in distances, angles and torsion angles; correlations between e.s.d.'s in cell parameters are only used when they are defined by crystal symmetry. An approximate (isotropic) treatment of cell e.s.d.'s is used for estimating e.s.d.'s involving l.s. planes.
Refinement. Refinement of *F*^2^ against ALL reflections. The weighted *R*-factor *wR* and goodness of fit *S* are based on *F*^2^, conventional *R*-factors *R* are based on *F*, with *F* set to zero for negative *F*^2^. The threshold expression of *F*^2^ > σ(*F*^2^) is used only for calculating *R*-factors(gt) *etc*. and is not relevant to the choice of reflections for refinement. *R*-factors based on *F*^2^ are statistically about twice as large as those based on *F*, and *R*- factors based on ALL data will be even larger.

### Fractional atomic coordinates and isotropic or equivalent isotropic displacement parameters (Å^2^)

**Table:**

	*x*	*y*	*z*	*U*_iso_*/*U*_eq_	
Mn1	0.5000	0.26907 (4)	0.2500	0.0319 (2)	
N1	0.41792 (13)	0.16367 (15)	0.2907 (3)	0.0378 (6)	
N2	0.27367 (13)	0.16204 (14)	−0.1202 (2)	0.0341 (6)	
N3	0.32528 (13)	0.25285 (15)	−0.2755 (2)	0.0352 (6)	
N4	0.39898 (13)	0.23967 (15)	0.0092 (2)	0.0319 (5)	
N5	0.44191 (14)	0.33368 (17)	−0.1419 (3)	0.0506 (7)	
H5A	0.4366	0.3567	−0.2322	0.061*	
H5B	0.4825	0.3490	−0.0548	0.061*	
N6	0.20573 (14)	0.17337 (17)	−0.3926 (3)	0.0517 (8)	
H6A	0.2000	0.1938	−0.4847	0.062*	
H6B	0.1697	0.1376	−0.3858	0.062*	
N7	0.56500 (14)	0.37854 (17)	0.1881 (3)	0.0400 (6)	
N8	0.58818 (14)	0.44325 (19)	0.2785 (3)	0.0420 (6)	
N9	0.61109 (17)	0.50489 (19)	0.3684 (3)	0.0631 (9)	
C1	0.4266 (2)	0.1298 (2)	0.4336 (4)	0.0555 (9)	
H1A	0.4746	0.1416	0.5230	0.067*	
C2	0.3687 (2)	0.0789 (2)	0.4542 (4)	0.0595 (10)	
H2A	0.3773	0.0572	0.5555	0.071*	
C3	0.29800 (19)	0.0605 (2)	0.3237 (4)	0.0478 (8)	
H3A	0.2576	0.0263	0.3350	0.057*	
C4	0.28749 (17)	0.09327 (18)	0.1750 (3)	0.0385 (7)	
H4A	0.2403	0.0807	0.0843	0.046*	
C5	0.34803 (16)	0.14502 (18)	0.1631 (3)	0.0306 (6)	
C6	0.33940 (16)	0.18485 (17)	0.0066 (3)	0.0291 (6)	
C7	0.26999 (16)	0.19689 (19)	−0.2612 (3)	0.0341 (7)	
C8	0.38731 (16)	0.27449 (19)	−0.1370 (3)	0.0329 (6)	

### Atomic displacement parameters (Å^2^)

**Table:**

	*U*^11^	*U*^22^	*U*^33^	*U*^12^	*U*^13^	*U*^23^
Mn1	0.0284 (3)	0.0374 (4)	0.0248 (3)	0.000	0.0064 (2)	0.000
N1	0.0369 (14)	0.0401 (15)	0.0285 (13)	−0.0048 (11)	0.0062 (11)	0.0062 (10)
N2	0.0321 (13)	0.0406 (14)	0.0254 (12)	−0.0039 (11)	0.0081 (10)	0.0001 (10)
N3	0.0322 (13)	0.0442 (15)	0.0243 (12)	−0.0066 (11)	0.0073 (10)	−0.0001 (10)
N4	0.0304 (13)	0.0382 (14)	0.0243 (11)	−0.0043 (11)	0.0087 (10)	0.0007 (10)
N5	0.0422 (16)	0.076 (2)	0.0260 (13)	−0.0219 (14)	0.0071 (11)	0.0089 (12)
N6	0.0431 (16)	0.076 (2)	0.0263 (13)	−0.0227 (14)	0.0057 (12)	0.0010 (12)
N7	0.0357 (15)	0.0461 (16)	0.0370 (14)	−0.0042 (12)	0.0142 (12)	−0.0013 (12)
N8	0.0308 (14)	0.0508 (17)	0.0409 (15)	−0.0008 (13)	0.0119 (12)	0.0098 (13)
N9	0.069 (2)	0.0530 (19)	0.0545 (18)	−0.0183 (15)	0.0141 (16)	−0.0114 (14)
C1	0.053 (2)	0.063 (2)	0.0345 (18)	−0.0142 (17)	0.0040 (15)	0.0131 (15)
C2	0.070 (2)	0.064 (2)	0.0374 (18)	−0.0201 (19)	0.0166 (17)	0.0160 (16)
C3	0.054 (2)	0.0431 (19)	0.052 (2)	−0.0071 (16)	0.0276 (17)	0.0071 (15)
C4	0.0367 (18)	0.0364 (17)	0.0383 (16)	−0.0056 (13)	0.0118 (14)	−0.0016 (13)
C5	0.0336 (16)	0.0296 (15)	0.0284 (14)	−0.0004 (12)	0.0127 (12)	0.0009 (12)
C6	0.0303 (15)	0.0286 (15)	0.0273 (14)	0.0015 (12)	0.0111 (12)	−0.0009 (11)
C7	0.0287 (16)	0.0393 (17)	0.0286 (15)	0.0007 (13)	0.0067 (12)	−0.0020 (12)
C8	0.0298 (15)	0.0406 (17)	0.0264 (14)	−0.0005 (13)	0.0100 (12)	0.0007 (12)

### Geometric parameters (Å, º)

**Table:**

Mn1—N7^i^	2.192 (2)	N5—H5B	0.8600
Mn1—N7	2.192 (2)	N6—C7	1.325 (3)
Mn1—N4	2.244 (2)	N6—H6A	0.8600
Mn1—N4^i^	2.244 (2)	N6—H6B	0.8600
Mn1—N1	2.277 (2)	N7—N8	1.201 (3)
Mn1—N1^i^	2.277 (2)	N8—N9	1.164 (3)
N1—C1	1.346 (3)	C1—C2	1.368 (4)
N1—C5	1.348 (3)	C1—H1A	0.9300
N2—C6	1.315 (3)	C2—C3	1.369 (4)
N2—C7	1.365 (3)	C2—H2A	0.9300
N3—C8	1.336 (3)	C3—C4	1.380 (4)
N3—C7	1.345 (3)	C3—H3A	0.9300
N4—C6	1.340 (3)	C4—C5	1.379 (4)
N4—C8	1.364 (3)	C4—H4A	0.9300
N5—C8	1.330 (3)	C5—C6	1.493 (3)
N5—H5A	0.8600		
			
N7^i^—Mn1—N7	87.95 (13)	H6A—N6—H6B	120.0
N7^i^—Mn1—N4	94.56 (8)	N8—N7—Mn1	116.8 (2)
N7—Mn1—N4	101.08 (8)	N9—N8—N7	178.8 (3)
N7^i^—Mn1—N4^i^	101.08 (8)	N1—C1—C2	123.4 (3)
N7—Mn1—N4^i^	94.56 (8)	N1—C1—H1A	118.3
N4—Mn1—N4^i^	158.23 (11)	C2—C1—H1A	118.3
N7^i^—Mn1—N1	88.17 (9)	C1—C2—C3	119.0 (3)
N7—Mn1—N1	172.72 (8)	C1—C2—H2A	120.5
N4—Mn1—N1	73.10 (8)	C3—C2—H2A	120.5
N4^i^—Mn1—N1	92.23 (8)	C2—C3—C4	119.0 (3)
N7^i^—Mn1—N1^i^	172.72 (8)	C2—C3—H3A	120.5
N7—Mn1—N1^i^	88.17 (9)	C4—C3—H3A	120.5
N4—Mn1—N1^i^	92.23 (8)	C5—C4—C3	119.0 (3)
N4^i^—Mn1—N1^i^	73.10 (8)	C5—C4—H4A	120.5
N1—Mn1—N1^i^	96.33 (12)	C3—C4—H4A	120.5
C1—N1—C5	117.0 (2)	N1—C5—C4	122.6 (2)
C1—N1—Mn1	126.03 (19)	N1—C5—C6	115.8 (2)
C5—N1—Mn1	116.15 (17)	C4—C5—C6	121.6 (2)
C6—N2—C7	114.1 (2)	N2—C6—N4	126.7 (2)
C8—N3—C7	114.8 (2)	N2—C6—C5	116.2 (2)
C6—N4—C8	114.4 (2)	N4—C6—C5	117.1 (2)
C6—N4—Mn1	117.05 (16)	N6—C7—N3	118.7 (3)
C8—N4—Mn1	128.46 (18)	N6—C7—N2	116.0 (3)
C8—N5—H5A	120.0	N3—C7—N2	125.3 (2)
C8—N5—H5B	120.0	N5—C8—N3	117.8 (2)
H5A—N5—H5B	120.0	N5—C8—N4	117.6 (2)
C7—N6—H6A	120.0	N3—C8—N4	124.6 (3)
C7—N6—H6B	120.0		
			
N7^i^—Mn1—N1—C1	−81.4 (3)	C1—N1—C5—C4	−0.4 (4)
N4—Mn1—N1—C1	−176.8 (3)	Mn1—N1—C5—C4	−170.5 (2)
N4^i^—Mn1—N1—C1	19.6 (3)	C1—N1—C5—C6	178.9 (3)
N1^i^—Mn1—N1—C1	92.8 (3)	Mn1—N1—C5—C6	8.9 (3)
N7^i^—Mn1—N1—C5	87.6 (2)	C3—C4—C5—N1	1.2 (4)
N4—Mn1—N1—C5	−7.71 (19)	C3—C4—C5—C6	−178.2 (3)
N4^i^—Mn1—N1—C5	−171.4 (2)	C7—N2—C6—N4	2.4 (4)
N1^i^—Mn1—N1—C5	−98.1 (2)	C7—N2—C6—C5	−177.4 (2)
N7^i^—Mn1—N4—C6	−81.2 (2)	C8—N4—C6—N2	0.7 (4)
N7—Mn1—N4—C6	−170.01 (19)	Mn1—N4—C6—N2	177.3 (2)
N4^i^—Mn1—N4—C6	54.83 (18)	C8—N4—C6—C5	−179.5 (2)
N1—Mn1—N4—C6	5.48 (18)	Mn1—N4—C6—C5	−2.9 (3)
N1^i^—Mn1—N4—C6	101.42 (19)	N1—C5—C6—N2	175.8 (2)
N7^i^—Mn1—N4—C8	94.8 (2)	C4—C5—C6—N2	−4.9 (4)
N7—Mn1—N4—C8	6.0 (2)	N1—C5—C6—N4	−4.1 (4)
N4^i^—Mn1—N4—C8	−129.1 (2)	C4—C5—C6—N4	175.3 (2)
N1—Mn1—N4—C8	−178.5 (2)	C8—N3—C7—N6	178.8 (3)
N1^i^—Mn1—N4—C8	−82.5 (2)	C8—N3—C7—N2	−0.5 (4)
N7^i^—Mn1—N7—N8	43.16 (18)	C6—N2—C7—N6	178.2 (2)
N4—Mn1—N7—N8	137.4 (2)	C6—N2—C7—N3	−2.6 (4)
N4^i^—Mn1—N7—N8	−57.8 (2)	C7—N3—C8—N5	−176.4 (3)
N1^i^—Mn1—N7—N8	−130.7 (2)	C7—N3—C8—N4	4.1 (4)
C5—N1—C1—C2	−0.3 (5)	C6—N4—C8—N5	176.3 (2)
Mn1—N1—C1—C2	168.6 (3)	Mn1—N4—C8—N5	0.1 (4)
N1—C1—C2—C3	0.3 (6)	C6—N4—C8—N3	−4.2 (4)
C1—C2—C3—C4	0.4 (5)	Mn1—N4—C8—N3	179.68 (19)
C2—C3—C4—C5	−1.1 (5)		

Symmetry code: (i) −*x*+1, *y*, −*z*+1/2.

### Hydrogen-bond geometry (Å, º)

**Table:**

*D*—H···*A*	*D*—H	H···*A*	*D*···*A*	*D*—H···*A*
N5—H5*B*···N7	0.86	2.14	2.986 (3)	166
N5—H5*A*···N9^ii^	0.86	2.32	2.996 (4)	136
N6—H6*A*···N3^iii^	0.86	2.19	3.048 (3)	175
N6—H6*B*···N7^iv^	0.86	2.30	3.063 (3)	148

Symmetry codes: (ii) −*x*+1, −*y*+1, −*z*; (iii) −*x*+1/2, −*y*+1/2, −*z*−1; (iv) *x*−1/2, −*y*+1/2, *z*−1/2.
